# Supplementary material for: Sexual Dimorphism of Skeletal Muscle in a Mouse Model of Breast Cancer: A Functional and Molecular Analysis
Source: Int J Mol Sci. 2023 Jul 19;24(14):11669. doi: 10.3390/ijms241411669 (PMC10380440; doi:10.3390/ijms241411669)
Supplement: Supplementary file 1 [file ijms-24-11669-s001.zip › ijms-2501158-supplementary.pdf]

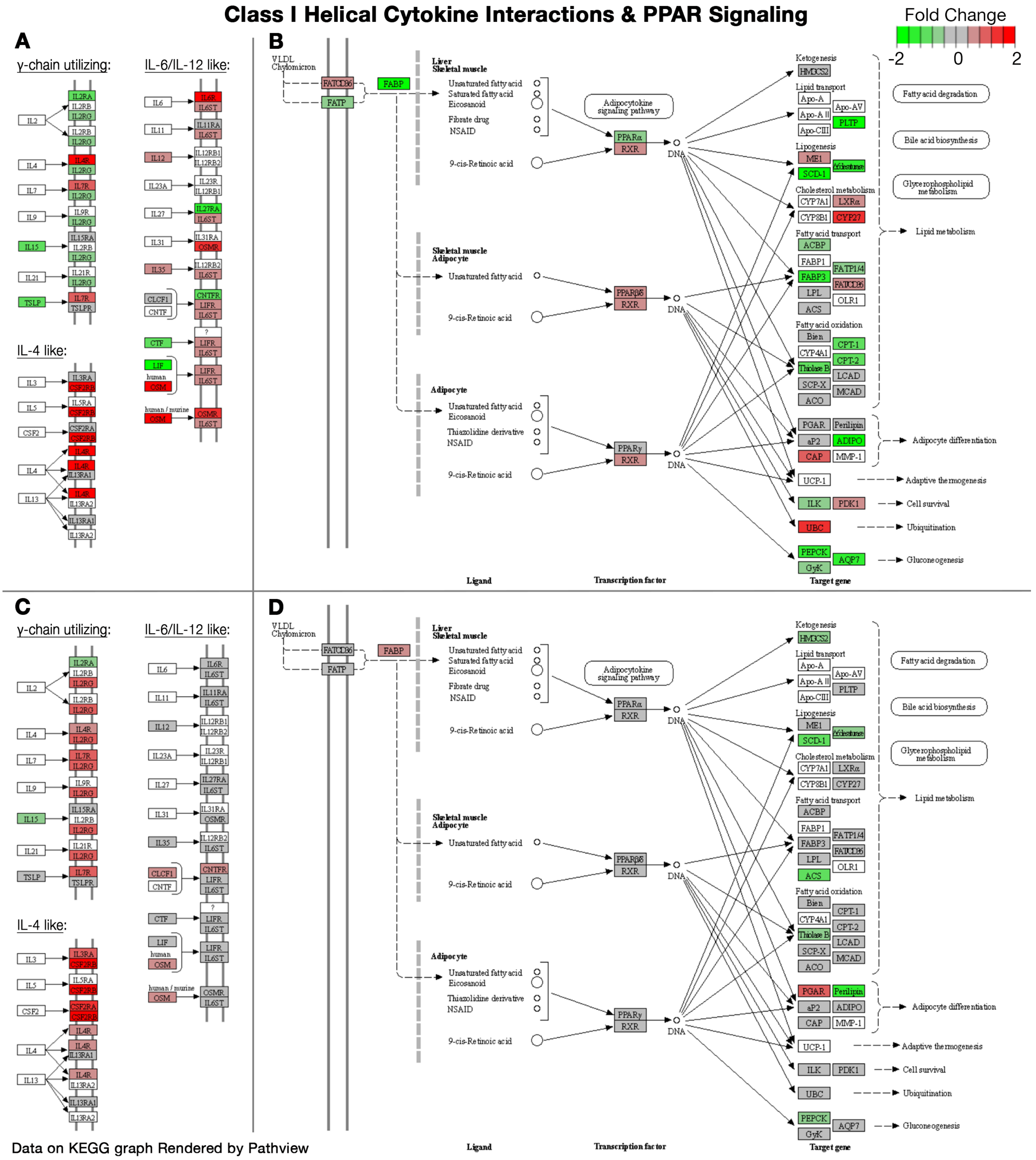

Figure S1. Differential Pathway Expression of Class I Helical Cytokine Interactions and PPAR Signaling

Figure modified from KEGG graphs rendered by Pathview. (**A**) Differential expression in female tumor mice compared to female controls in the Class I Helical Cytokine Interactions. (**B**) Differential expression in female tumor mice compared to female controls in the PPAR signaling pathway. (**C**) Differential expression in male tumor mice compared to male controls in the Class I Helical Cytokine Interactions. (**D**) Differential expression in male tumor mice compared to male controls in the PPAR signaling pathway. RNA sequencing was performed using an *n*=5 mice for each sample group.

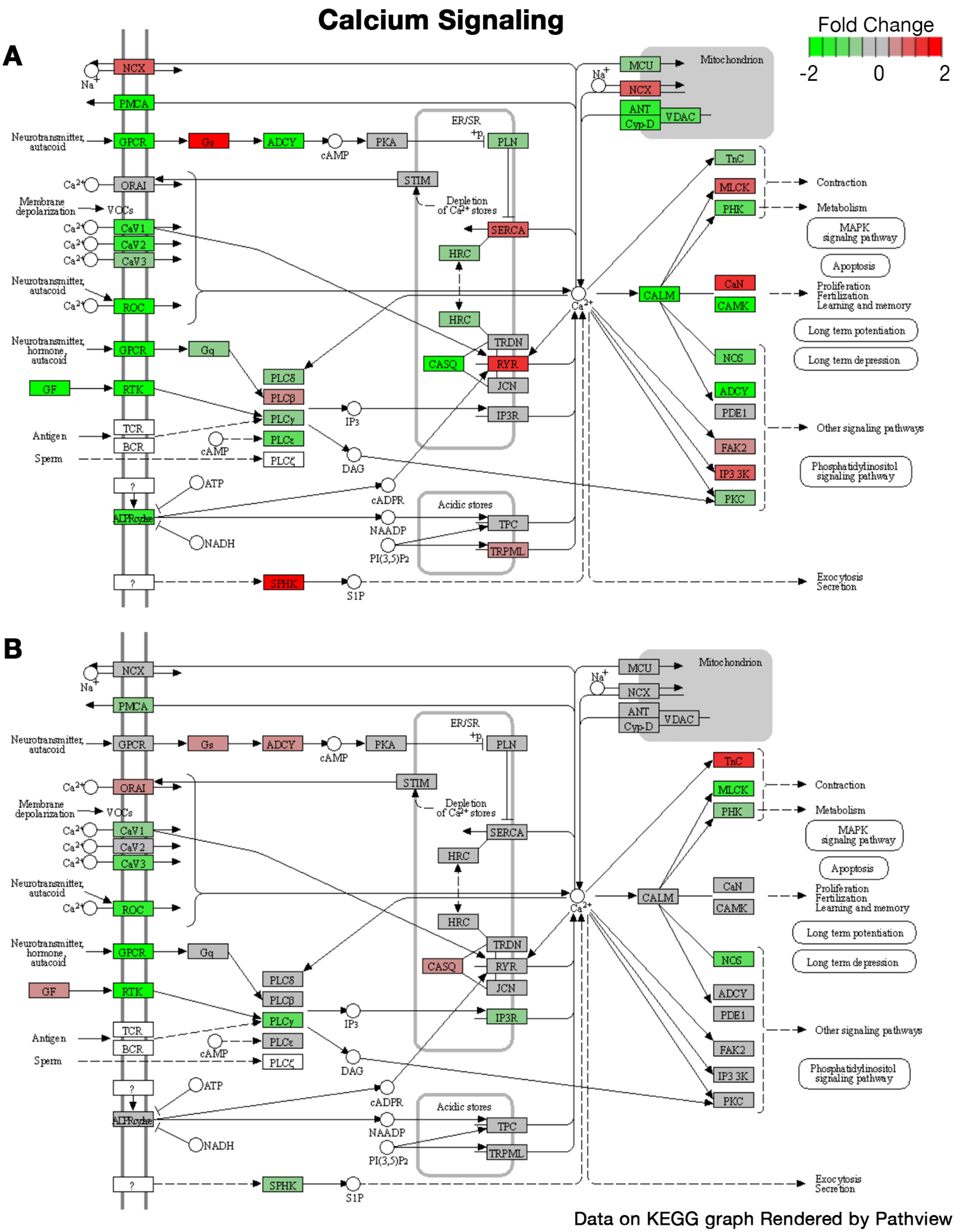

Figure S2. Differential Pathway Expression of Calcium Signaling Pathway

Figure modified from KEGG graphs rendered by Pathview. (**A**) Differential expression in female tumor mice compared to female controls. (**B**) Differential expression in male tumor mice compared to male controls. RNA sequencing was performed using an *n*=5 mice for each sample group.

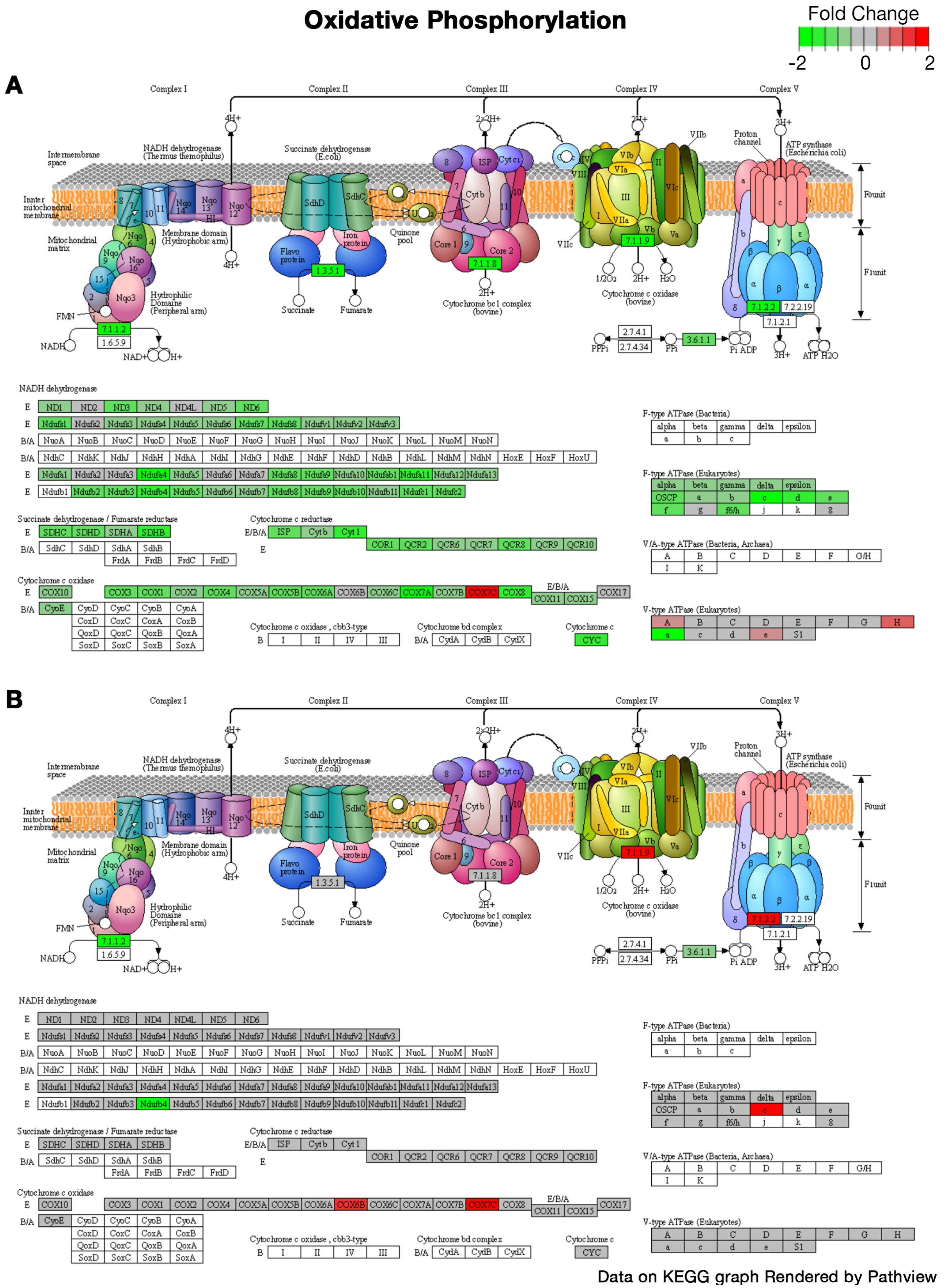

Figure S3. Differential Pathway Expression of Oxidative Phosphorylation Pathway

Figure modified from KEGG graphs rendered by Pathview. (**A**) Differential expression in female tumor mice compared to female controls. (**B**) Differential expression in male tumor mice compared to male controls. RNA sequencing was performed using an *n*=5 mice for each sample group.

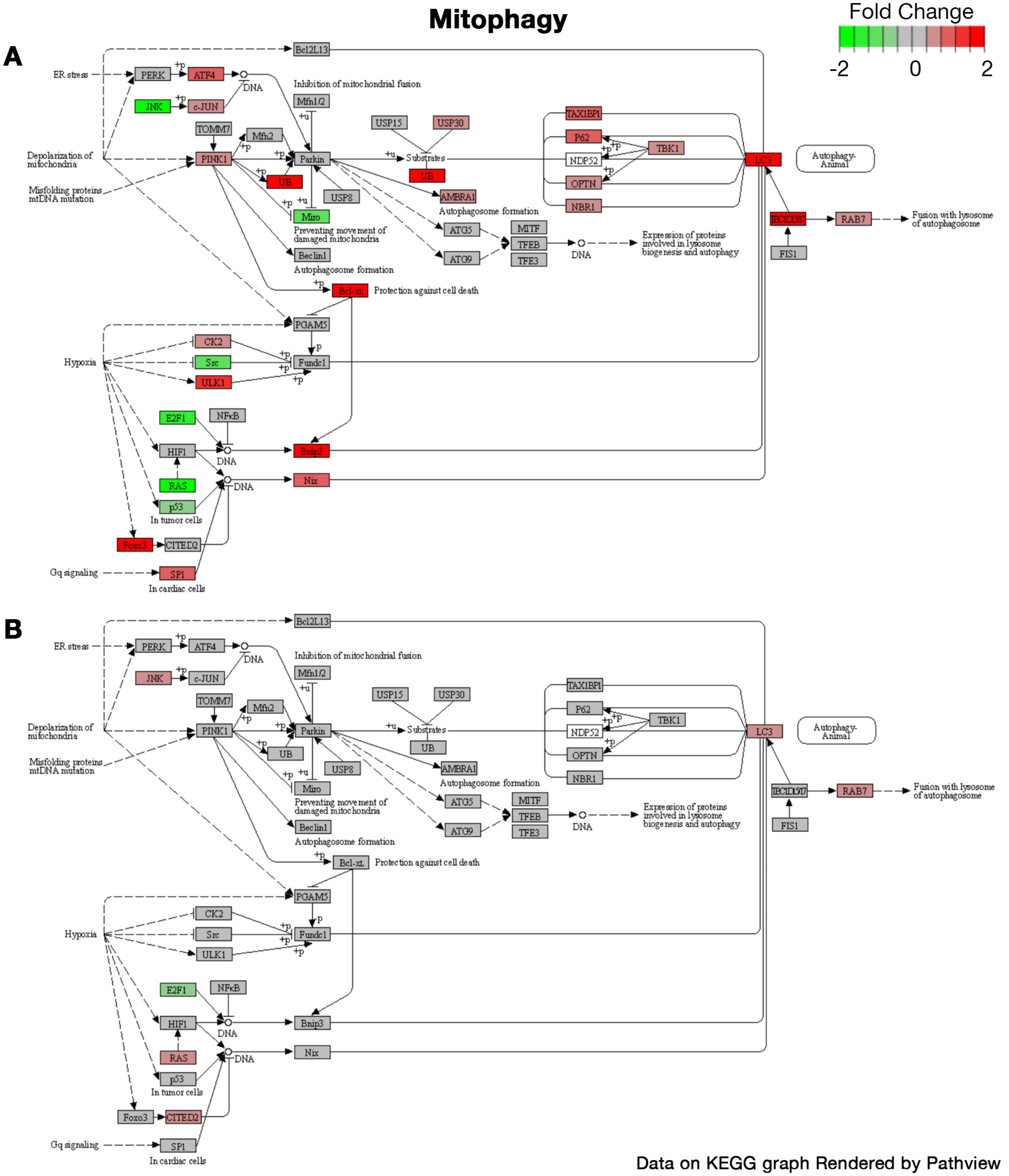

Figure S4. Differential Pathway Expression of Mitophagy Pathway

Figure modified from KEGG graphs rendered by Pathview. (**A**) Differential expression in female tumor mice compared to female controls. (**B**) Differential expression in male tumor mice compared to male controls. RNA sequencing was performed using an *n*=5 mice for each sample group.

Table S1. Sexually Dimorphic Gene Expression in Control Mice

Gene	Gene Name	Log_2 Fold Change	Adj. *p*-value
Aldh1a1	Aldehyde dehydrogenase 1 family, member A1	0.7367	0.0037
Cd24a	Cluster of differentiation 24/heat stable antigen	1.0911	9.49E-08
Ddx3y	DEAD-Box helicase 3, Y-linked	12.1534	3.46E-36
Grb10	Growth factor receptor bound protein 10	-1.0939	7.5E-11
Irx3	Iroquois homeobox 3	0.25540	0.42151
Kdm5d	Lysine (K)-specific demethylase 5D	10.2048	2.11E-25
Kdm6a	Lysine (K)-specific demethylase 6A	-0.4411	0.05701
Oas2	2'-5'-oligoadenylate synthetase 2	-0.99802	0.03672
Rps4x	Ribosomal protein S4, X-linked	0.07661	0.71415
Wwp1	WW Domain containing E3 Ubiquitin Protein Ligase 1	1.62777	3.84E-18
Xist	Inactive X-specific transcript	-12.3299	1.33E-89

Fold change and significance data for differential expression between male controls and female controls in genes previously identified as being sexually dimorphic in mouse skeletal muscle. Positive and negative log_2 fold change values represent higher expression in male vs female controls, respectively. [adj, adjusted]

Table S2. Group Sample Size by Variable

	Variable	Study Group			
		FC	FT	MC	MT
RNA Sequencing	Gene Expression	5 (5)	5 (5)	5 (5)	5 (5)
Weights	TA	16 (8)	10 (5)	19 (10)	18 (9)
	EDL	16 (8)	10 (5)	19 (10)	18 (9)
	Gastrocnemius	16 (8)	10 (5)	19 (10)	18 (9)
	Soleus	16 (8)	10 (5)	19 (10)	18 (9)
	Body Mass	16 (8)	10 (5)	19 (10)	18 (9)
Muscle Contractile Properties	EDL Weight	8 (8)	5 (5)	5 (5)	12 (7)
	L_o	8 (8)	5 (5)	5 (5)	11 (7)
	CSA	8 (8)	5 (5)	5 (5)	11 (7)
	Peak Twitch	8 (8)	5 (5)	5 (5)	12 (7)
	Peak CT	8 (8)	5 (5)	5 (5)	12 (7)
	Peak RFD	8 (8)	5 (5)	5 (5)	12 (7)
	Peak ½ RT	8 (8)	5 (5)	5 (5)	12 (7)
	Peak RR	8 (8)	5 (5)	5 (5)	12 (7)
	Peak Tetanus	8 (8)	5 (5)	5 (5)	7 (6)
	Twitch/CSA	8 (8)	5 (5)	5 (5)	11 (7)
	Tetanus/CSA	8 (8)	5 (5)	5 (5)	7 (6)
	Twitch/Tetanus	8 (8)	5 (5)	5 (5)	7 (6)
Force-Frequency	Force (@ each frequency)	8 (8)	5 (5)	5 (5)	7 (6)
Fatigue	Force (@ each repetition)	8 (8)	5 (5)	5 (5)	12 (7)

Group sample sizes for each variable are presented as: # of measurements (# of mice); this considers inter-sample measures from one or both legs from a given mouse. [CSA, cross sectional area; CT, contraction time; EDL, extensor digitorum longus; FC, female control group; FT, female tumor group; L_o, optimal length; MC, male control group; MT, male tumor group; RFD, rate of force development; RR, relaxation rate; RT, relaxation time; TA, tibialis anterior]

Table S3. Tumor-Induced and Sexual Dimorphic Effects on EDL Fatiguability

Fixed Effect	Absolute Force		Relative Force	
	F Ratio	***p*-value**	F Ratio	***p*-value**
Sex	$F_{(1, 89.5)} = 5.69$	**0.0191**	$F_{(1, 156.5)} = 0.004$	0.9506
Group	$F_{(1, 89.5)} = 16.36$	**0.0002**	$F_{(1, 156.5)} = 0.004$	0.9506
Repetition	$F_{(359, 9339)} = 307.7$	**<0.0001**	$F_{(359, 9339)} = 812.4$	**<0.0001**
Sex*Group	$F_{(1, 89.5)} = 90.96$	**<0.0001**	$F_{(1, 156.5)} = 0.004$	0.9506
Sex*Repetition	$F_{(359, 9339)} = 1.099$	0.0992	$F_{(359, 9339)} = 7.582$	**<0.0001**
Group*Repetition	$F_{(359, 9339)} = 2.091$	**<0.0001**	$F_{(359, 9339)} = 1.806$	**<0.0001**
Sex*Group*Repetition	$F_{(359, 9339)} = 8.290$	**<0.0001**	$F_{(359, 9339)} = 1.002$	0.4783

Fixed effect statistics for linear mixed models assessing EDL fatiguability based upon sex (male vs female) and tumor group (control vs tumor) in a 360-repetition contraction protocol. Absolute forces represent net forces produced by the muscle, whereas relative forces are reflective of the percent change in force from the first repetition. Models were run with main effects of sex, group, and repetition, as well as all combinations of interactions among the main effects.

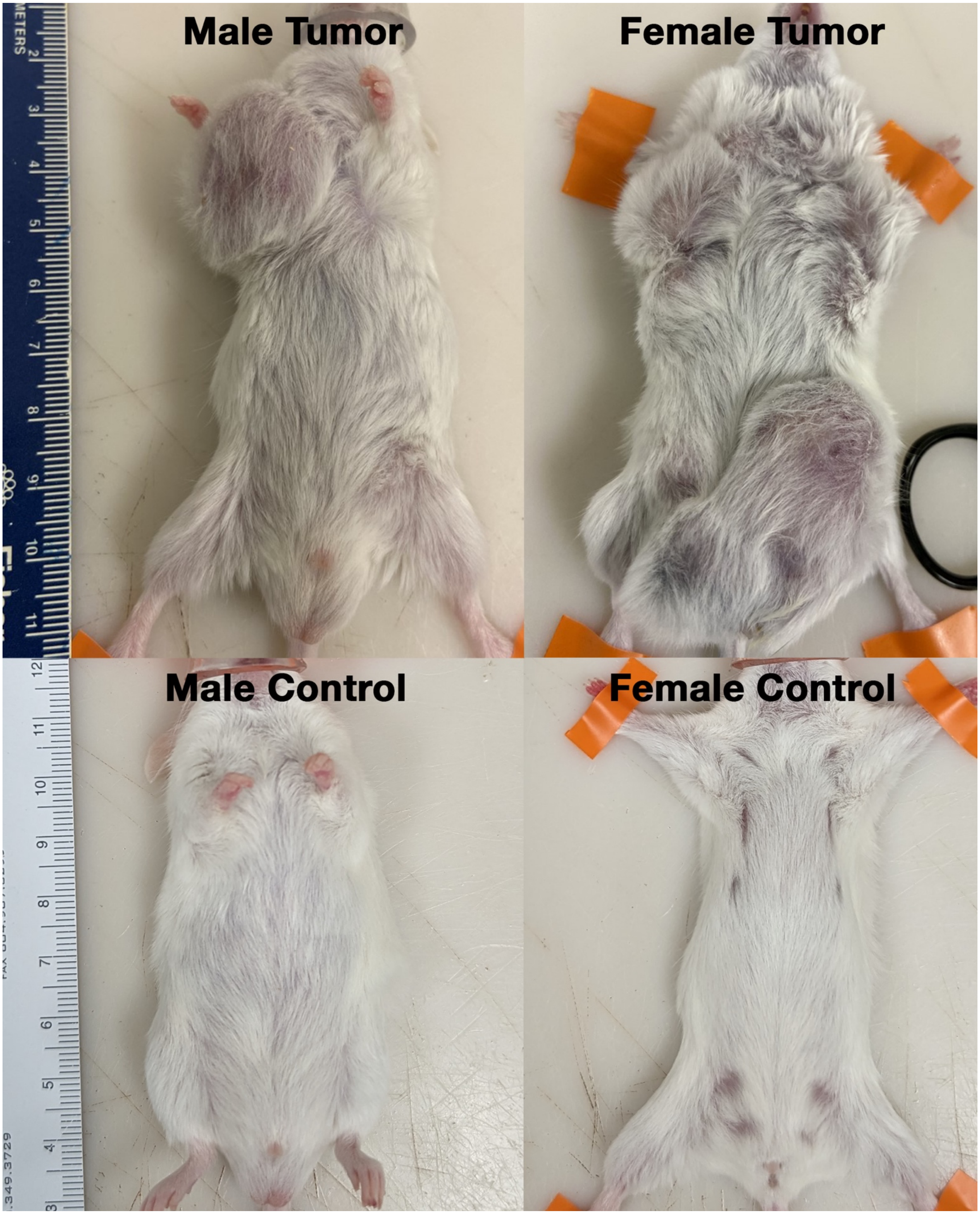

Figure S5. Representative Images of Mice at Time of Euthanasia
Representation of tumor burden in male and female tumor mice, as well as their respective age matched controls, at time of euthanasia. A ruler (in centimeters) was placed for size reference.
